# Identifying neurodevelopmental anomalies of white matter microstructure associated with high risk for psychosis in 22q11.2DS

**DOI:** 10.1038/s41398-020-01090-z

**Published:** 2020-11-24

**Authors:** Joëlle Bagautdinova, Maria C. Padula, Daniela Zöller, Corrado Sandini, Maude Schneider, Marie Schaer, Stephan Eliez

**Affiliations:** 1 Developmental Imaging and Psychopathology Laboratory, University of Geneva School of Medicine, Geneva, Switzerland; 2 Medical Image Processing Laboratory, Institute of Bioengineering, École Polytechnique Fédérale de Lausanne (EPFL), Lausanne, Switzerland; 3 Department of Radiology and Medical Informatics, University of Geneva, Geneva, Switzerland; 4 Institute of Neuromodulation and Neurotechnology, Department of Neurosurgery and Neurotechnology, University of Tübingen, Tübingen, Germany; 5 Clinical Psychology Unit for Intellectual and Developmental Disabilities, Faculty of Psychology and Educational Sciences, University of Geneva, Geneva, Switzerland

**Keywords:** Neuroscience, Predictive markers, Schizophrenia

## Abstract

Disruptions of white matter microstructure have been widely reported in schizophrenia. However, the emergence of these alterations during preclinical stages remains poorly understood. 22q11.2 Deletion Syndrome (22q11.2DS) represents a unique model to study the interplay of different risk factors that may impact neurodevelopment in premorbid psychosis. To identify the impact of genetic predisposition for psychosis on white matter development, we acquired longitudinal MRI data in 201 individuals (22q11.2DS = 101; controls = 100) aged 5–35 years with 1–3 time points and reconstructed 18 white matter tracts using TRACULA. Mixed model regression was used to characterize developmental trajectories of four diffusion measures—fractional anisotropy (FA), axial (AD), radial (RD), and mean diffusivity (MD) in each tract. To disentangle the impact of additional environmental and developmental risk factors on white matter maturation, we used a multivariate approach (partial least squares (PLS) correlation) in a subset of 39 individuals with 22q11.2DS. Results revealed no divergent white matter developmental trajectories in patients with 22q11.2DS compared to controls. However, 22q11.2DS showed consistently increased FA and reduced AD, RD, and MD in most white matter tracts. PLS correlation further revealed a significant white matter-clinical risk factors relationship. These results indicate that while age-related changes are preserved in 22q11.2DS, white matter microstructure is widely disrupted, suggesting that genetic high risk for psychosis involves early occurring neurodevelopmental insults. In addition, multivariate modeling showed that clinical risk factors further impact white matter development. Together, these findings suggest that genetic, developmental, and environmental risk factors may play a cumulative role in altering normative white matter development during premorbid stages of psychosis.

## Introduction

Schizophrenia is a severe chronic psychiatric disorder affecting ~1% of the world population and involving a very high societal cost^1^. While extensive research has been performed to uncover the etiological mechanisms of the illness, the complexity and heterogeneity of the disorder have made this extremely challenging. It has become increasingly clear, however, that schizophrenia is a progressive neurodevelopmental disorder prompted by the cumulation of genetic, developmental and environmental “hits” that collectively alter the normal course of brain maturation^2–4^. Studying the developmental stages preceding the emergence of psychosis is therefore key if we are to unravel the etiological pathways of the disorder. A better understanding of the neuropathological substrates underlying schizophrenia may, in turn, provide important markers for early detection and targets for intervention, which could significantly improve the outcome of patients^2,4^ and prevent a downward cascade towards a full-blown psychosis.

Evidence from genetic, postmortem, and neuroimaging studies have increasingly pointed to disrupted white matter structure and function as central neuropathological mechanisms in psychosis^5^, supporting the “dysconnectivity theory” of schizophrenia^6,7^. More specifically, anomalies of white matter structure have been shown to undergo a progressive aggravation as the illness progresses from preclinical to chronic stages^8,9^, suggesting an involvement of aberrant white matter development in the neuropathology of schizophrenia. However, research on the early phases of the trajectory to psychosis has been limited by the fact that individuals are mostly identified at the onset of psychosis.

Genetic syndromes represent a unique opportunity to study the interplay of different risk factors involved in the unfolding of psychosis during development, as syndromic individuals carry a homogenous risk for the illness and are usually diagnosed early in life. One of the strongest genetic risk factors for psychosis is 22q11.2 deletion syndrome (22q11.2DS)^10^, a neurogenetic developmental disorder occurring with a prevalence of one in 2000–4000 live births^11^ and with a conversion rate to psychosis of around 40%^12^. Neuroimaging studies have consistently reported extensive structural and functional brain alterations in the syndrome, and involve thicker cortex and reduced surface area^13^, reduced whole-brain volume and gyrification, as well as decreased functional connectivity (for a review, see ref. ^14^), affecting predominantly frontal and posterior brain regions^15^. Furthermore, similar to the findings on schizophrenia reported above, volumetric reductions^16–18^, widespread alterations of white matter microstructure^19^, and anomalies of white matter development^20,21^ have also been widely reported in 22q11.2DS, suggesting the involvement of a common white matter pathology. However, due to the relatively small size of these studies, the direction of alterations varied considerably and prevented a clear identification of disruptions associated with 22q11.2DS^15,19^. A critical advance on white matter alterations in 22q11.2DS was recently made by a large-scale cross-sectional multisite study, which revealed a more consistent pattern of alterations in syndromic individuals involving reduced diffusivity and a combination of increased and decreased anisotropy^22^. The study further reported older age at white matter maturation peak in 22q11.2DS, suggesting delayed white matter development. However, the cross-sectional nature of neuroimaging studies performed so far in 22q11.2DS may be suboptimal to accurately delineate trajectories of white matter development, as they do not account for within-subject change^23^. This is even more likely given that longitudinal studies on typically developing individuals have shown complex, non-linear developmental curves of white matter maturation extending through adulthood^24–26^. Therefore, a longitudinal delineation of white matter development in 22q11.2DS is needed to clarify the maturational profile of white matter microstructure related to genetic risk for psychosis.

While genetic factors such as the 22q11.2 deletion can account for an initial vulnerability to psychosis, additional insults occurring during infancy, childhood, and/or adolescence may play a cumulative role in triggering psychosis^2,4,27^. In 22q11.2DS, it has been shown that individuals who develop schizophrenia typically present a lower IQ^28,29^ and display a cognitive decline starting from 11 years^28^. Similarly, other studies showed that patients with 22q11.2DS born before term (<37 weeks of gestation)^30,31^ or presenting an anxiety disorder at their baseline assessment^29^ are more likely to develop schizophrenia. Finally, the ultra high-risk (UHR) status (i.e., presence of attenuated positive symptoms (APS), brief intermittent psychotic syndrome (BIPS), and genetic risk plus recent deterioration (GRD))^32^ has also been identified as a strong predictor of psychosis in these patients^33^.

Apart from univariate studies that investigated white matter microstructure in patients with 22q11.2DS presenting psychotic symptoms^19,34–36^ or a cognitive decline^37^, the impact of the above-mentioned risk factors on white matter maturation remains largely unknown. Evidence increasingly shows that white matter alterations associated with psychosis are widespread and subtle^38,39^, suggesting that multivariate approaches may be better suited to capture the complex nature of brain alterations related to premorbid stages. Given that prediction of conversion based solely on clinical markers has proven limited success^40^, the addition of such multivariate neuroimaging-based biomarkers has the potential to significantly improve the early detection of psychosis.

Thus, to clarify the nature of white matter alterations in individuals at genetic high risk of psychosis, our first objective was to delineate the developmental trajectories of long-range white matter tracts’ microstructure in 22q11.2DS using a longitudinal design involving repeated diffusion tensor imaging (DTI) assessments per participant and mixed model regression^41^. Based on previous findings reported in cross-sectional studies, we hypothesized that the genetic risk conveyed by 22q11.2DS would be associated with widespread alterations in DTI metrics and abnormal age-related changes. Our second objective was to determine whether identified clinical risk factors of psychosis (i.e., UHR status, low cognitive functioning at baseline, cognitive decline, preterm birth, and the presence of an anxiety disorder at baseline) were associated with specific patterns of brain alterations. We used partial least squares (PLS) correlation^42^, a multivariate approach specifically tailored to capture complex patterns of association between clinical and brain measures. We hypothesized that clinical risk factors of psychosis would be associated with a widespread pattern of white matter alterations affecting multiple tracts and diffusion measures, reflecting a specific effect of additional developmental and environmental risk factors on white matter maturation.

## Materials and methods

### Participants

The study was approved by the Geneva Ethics Committee. Written informed consent was given by the participants and their parents. Individuals with 22q11.2DS and controls were recruited in the context of an ongoing longitudinal study, using parent associations or through word of mouth.

Two distinct analyses were conducted in this study. First, for the longitudinal characterization of white matter development in 22q11.2DS compared to controls, the sample consisted of 201 participants (*N* = 100 controls (48 males), *N* = 101 22q11.2DS (51 males)) aged 5–35 years who contributed a total of 302 scans (*N* = 166 22q11.2DS, *N* = 136 control scans) (Supplementary Figure S1A). For detailed demographic information, see Supplementary Table S1. Second, for the multivariate PLS correlation analysis assessing the impact of clinical risk factors on white matter development, the analyses were performed on a subset of 39 patients with 22q11.2DS aged 10–29 years who had multiple visits and information regarding all five risk factors (for a description of clinical and cognitive assessments used to determine the presence of risk factors, see below), resulting in a subset of 88 scans (Supplementary Figure S1B).

### Medical, cognitive, and psychiatric assessment

Pregnancy duration was assessed using a medical questionnaire completed by the parents of patients with 22q11.2DS. Preterm birth was established when pregnancy duration was <37 weeks. Full-scale IQ (FSIQ) was evaluated in all participants using age-adapted versions of the Wechsler intelligence scale (i.e., the Wechsler Intelligence Scale for Children, version III or IV, or the Wechsler Adult Intelligence Scale, version III or IV)^43–46^. Psychiatric assessment was performed using the Diagnostic Interview for Children and Adolescents Revised (DICA-R)^47^, the psychosis supplement from the Kiddie-Schedule for Affective Disorders and Schizophrenia Present and Lifetime version (K-SADS-PL)^48^, and the Structured Clinical Interview for DSM-IV Axis I Disorders (SCID-I)^49^ for adult patients (starting from 18 years). The Structured Interview for Psychosis-Risk Syndromes (SIPS)^50^ was used to assess the UHR criteria i.e., attenuated positive symptoms (APS), brief intermittent psychotic syndrome (BIPS), and genetic risk plus recent deterioration (GRD) in individuals with 22q11.2DS starting from 10 years of age.

### Risk factors of psychosis

Information collected using the above-described assessment methods was subsequently used to define five dichotomized clinical risk factors of psychosis:

1. *Preterm birth*. Prematurity was established in patients born earlier than 37 weeks of pregnancy (*N* = 12 born preterm; *N* = 27 born at term).
2. *Low baseline FSIQ*. To dichotomize FSIQ at the first visit, we followed a similar approach to Vorstman and colleagues^28^ and divided the sample into two groups using FSIQ = 75 as the cut-off (*N* = 24 with FSIQ ≥ 75; *N* = 12 with FSIQ < 75).
3. *Cognitive decline*. The presence of a cognitive decline was determined as a negative difference between the latest and earliest FSIQ assessments; null or positive changes indicated an absence of cognitive degradation (*N* = 16 with cognitive decline; *N* = 23 without cognitive decline).
4. *Presence of an anxiety disorder at baseline*. Patients were considered at risk when they presented any type of anxiety disorder at their baseline evaluation (*N* = 21 with baseline anxiety disorder; *N* = 18 without baseline anxiety disorder). Subtypes of included anxiety disorders are described elsewhere^12^.
5. *UHR*. Patients belonged to the UHR category when they met the UHR criteria at least once during their development and, conversely, patients were considered non-UHR when they had never received a UHR diagnosis (*N* = 10 UHR; *N* = 29 non-UHR).

### MRI acquisitions, processing, and tractography

Structural T1-weighted and DTI images were acquired at the Center for Biomedical Imaging (CIBM) in Geneva, using a Siemens Trio (191 scans) or a Siemens Prisma (111 scans) 3 Tesla MRI scanner (see Supplementary Table S2 for the distribution of scans per group). Each scanner had a different head coil (12 channels for the Siemens Trio and 20 channels for the Siemens Prisma). The T1-weighted sequence was acquired with a 3D volumetric pulse, TR = 2500 ms, TE = 3 ms, flip angle = 8°, acquisition matrix = 256 × 256, field of view = 23.5 cm, slice thickness = 3.2 mm, 192 slices. DTI images parameters were as follows: number of directions = 30, *b* = 1000 s/mm^2^, TR = 8800 ms, TE = 84 ms, flip angle = 90°, acquisition matrix = 128 × 128, field of view = 25.6 cm, GRAPPA acceleration = 2, 64 axial slices, slice thickness = 2 mm.

T1-weighted structural scans were visually examined for motion artefacts and processed using the longitudinal pipeline of *FreeSurfer* v6.0 (http://surfer.nmr.mgh.harvard.edu). Briefly, the longitudinal stream uses all available scans of a given individual to create an unbiased within-subject template^51^. This method has been shown to increase reliability and statistical power, thereby improving the estimation of within-subject age-related changes^52^.

After visual inspection of DTI images for motion artefacts, we used the longitudinal pipeline of the TRacts Constrained by UnderLying Anatomy (TRACULA) tool^53^ from *FreeSurfer* v6.0 to reconstruct 18 major white matter paths in each subjects’ time points (for an example of tracts reconstruction performed by TRACULA, see Supplementary Figure S2). White matter tracts reconstructed by TRACULA are the following:

- Corpus callosum–forceps major (FMAJ) and forceps minor (FMIN)
- Anterior thalamic radiation (ATR)
- Corticospinal tract (CST)
- Superior longitudinal fasciculus–parietal bundle (SLFP)
- Superior longitudinal fasciculus–temporal bundle (SLFT)
- Inferior longitudinal fasciculus (ILF)
- Cingulum–cingulate gyrus bundle (CCG)
- Cingulum–angular bundle (CAB)
- Uncinate fasciculus (UNC)

The longitudinal framework of TRACULA is an automated global probabilistic tractography algorithm that estimates the probability distribution of white matter tracts given the T1-weighted and DTI information from all available time points. The probability distribution of a pathway is computed partly using the “ball-and-stick” model of diffusion, and partly using prior anatomical information. Importantly, the algorithm’s assumption is merely that tracts traverse the same anatomical regions, which is particularly relevant for clinical samples where tracts are likely to be somewhat altered in shape and location. Moreover, TRACULA performs tract reconstruction in the native space of the subject to ensure that the same white matter parts are compared between time points. This has been demonstrated to improve test-retest reliability and increase sensitivity to longitudinal changes in white matter tracts^53^, making it a particularly adapted tool for longitudinal studies of white matter development. Once the tracts distributions have been estimated, TRACULA extracts four diffusion measures as averages per tract: (1) fractional anisotropy (FA), indicating the fraction of diffusion that is directionally constrained; (2) axial diffusivity (AD), corresponding to the main direction of diffusion; radial diffusivity (RD), indicating the amount of diffusion perpendicular to the main direction; and mean diffusivity (MD), reflecting the magnitude of diffusivity in the tissue. Of note, tracts reconstruction was unsuccessful in three subjects (two patients with 22q11.2DS, one control), resulting in a sample of 199 subjects for the characterization of white matter development. See Supplementary Material (Method, Table S3) for a detailed description of image quality check, MRI processing steps, and head motion-related information.

### Statistical analyses

#### Mixed models regression analysis

Mixed models regression analyses were used to characterize the developmental trajectories of white matter pathways in 22q11.2DS and controls (based on the following toolbox: https://github.com/danizoeller/myMixedModelsTrajectories). This type of analysis has been applied in previous studies by our group with a similar longitudinal design^54–58^, as this approach is suitable for unbalanced data, i.e., with a broad age range at first visit, variable time intervals between assessments and a different number of visits per participant. Random-intercept models were fitted to the data (for a detailed description, see ref. ^55^). Within-subject variables were modeled as random effects, and population variables (diagnosis, age, and their interaction) were implemented as fixed effects. Normal distribution was verified in each model. Gender and scanner type were included as covariates. Different models (constant, linear, or quadratic) were fitted on each trajectory using the *nlmefit* function in MATLAB R2014b (MathWorks), and the best-fitting model was selected using the Bayesian information criterion (BIC). This process was performed for each tract and diffusion measure. *P*-values of group and interaction effects were estimated using a log-likelihood approach and corrected for multiple comparisons using the false discovery rate (FDR) method^59^ (significance level *p* < 0.05). Mixed models results contain two types of information: group effects, revealed by intercept differences; and age × group interaction effects, revealed by curve shape differences. Graphs display FDR-corrected *p*-values of group and interaction effects for best-model fits with 95% confidence interval error bands.

#### PLS correlation analysis

To detect white matter alterations associated with the presence of clinical risk factors of psychosis in patients with 22q11.2DS, we performed a PLS correlation analysis using in-house software running on MATLAB R2014b (MathWorks) (for a detailed description, see ref. ^60^; in-house scripts are based on a toolbox available online at https://github.com/danizoeller/myPLS). PLS correlation involves the computation of a correlation matrix **R** = **XY** between a set of subject-specific brain measures (i.e., white matter microstructure as measured through AD, RD, MD, and FA in the 18 reconstructed white matter tracts) (**X**) and behavioral measures (i.e., clinically-established risk factors) (**Y**). In the current study, a sample of 39 subjects with 22q11DS with 2–3 scans per subject (resulting in 88 scans) was selected for the PLS correlation analysis. To ensure that the PLS correlation analysis would not be merely capturing age-related maturation processes occurring in white matter tracts, but rather would capture potentially aberrant developmental patterns related to the presence of risk factors in selected individuals, we adopted a three-step approach to create the brain matrix (**X**): (1) First, mixed models computing the relationship between age and each brain measure in the group of all 39 patients with 22q11.2DS were estimated through the method described above and included gender and scanner type as covariates. This provided an average developmental curve for each tract and diffusion measure. As there are 18 tracts × 4 diffusion metrics, 72 models were fitted. (2) Given that PLS is not adapted for longitudinal data, we then computed the average diffusion metric for each white matter tract across the scans of each subject, resulting in 72 diffusion metrics per subject (one average measure for 18 tracts × 4 diffusion metrics). (3) Finally, in order to account for age, we extracted the residuals (i.e., the difference between the observed and predicted values of the models fitted in the first step) for all subjects in each of the 72 measures. These residuals can be considered as a summary measure indicating the deviation of a given subject with respect to the predicted development at corresponding ages and were used as brain measures. Thus, the resulting brain matrix (**X**) was a 39 (subjects) by 72 (white matter measures) matrix. Five dichotomized risk factors (UHR, baseline FSIQ, cognitive decline, preterm birth, and anxiety disorder at baseline) were entered in the matrix (**Y**), resulting in a 39 (subjects) by 5 (risk factors) matrix.

Then, **X** and **Y** were *z*-scored across subjects, and the correlation matrix **R** was computed through **R** = **Y**^**T**^**X**, resulting in a 5 (risk factors) by 72 (white matter measures) matrix containing the correlation between each risk factor and each white matter measure across subjects. The main correlation components were then extracted through singular value decomposition (SVD) of the correlation matrix **R** = **USV**. As **R** was a 5 × 72 matrix, a total of five components was extracted.

For each correlation component, the singular value (on the diagonal of **S**) reveals the amount of correlation explained by the component, while the behavior weights (columns of **U**) and brain weights (rows of **V**) indicate how strongly the original behavior and brain variables respectively contribute to the brain-behavior correlation. A schematic illustration of the procedure can be found in Supplementary Figure S3. Permutation testing using 1000 permutations was subsequently applied to determine whether a component explained a significant amount of correlation (*p* < 0.05). For each significant correlation component, a bootstrapping procedure of 500 random samples with replacement was applied to evaluate the robustness of the brain and behavior weights and provide a bootstrap distribution of the weight values. Bootstrap scores, computed by dividing the mean of the bootstrap distribution by its standard deviation, indicate the contribution of each original variable to the brain-behavior correlation and can be interpreted similarly to *z* scores. Therefore, in PLS the results, we highlighted tracts with bootstrap scores >1.96 or <−1.96, corresponding to a robustness at a confidence interval of 95%. Finally, a summary “brain score” **L**_**X**_ = **XV**^**T**^ was computed for each individual by projecting the original brain matrix **X** onto the brain weights **V**. Similarly, individual “behavior scores” **L**_**y**_ = **YU** were computed by projecting the original behavior matrix **Y** onto the behavior weights **U**. A higher brain/behavior score indicates that the individual’s brain/behavior pattern is close to the brain/behavior pattern captured by the correlation component. Pearson correlation was computed between individual brain (**L**_**x**_) and behavioral scores (**L**_**y**_) of participants to reflect the strength of the correlation captured by the correlation component.

## Results

### White matter developmental trajectories in 22q11.2DS versus controls

After correcting for multiple comparisons using the FDR method, mixed models comparing 22q11.2DS (*N* = 101) and controls (*N* = 100) revealed an absence of age × group interaction effects, indicating that both groups followed parallel developmental trajectories (i.e., of similar shape) in all tracts and diffusion measures. However, significant group (i.e., intercept) differences were found in all four diffusion measures and in most white matter tracts. Detailed model fits and *p*-values of group effects and of age × group interaction effects for each white matter tract and diffusion measure are reported in the Supplementary Table S4. Observed developmental trajectories and group differences are detailed below.

**Fig. 1:**
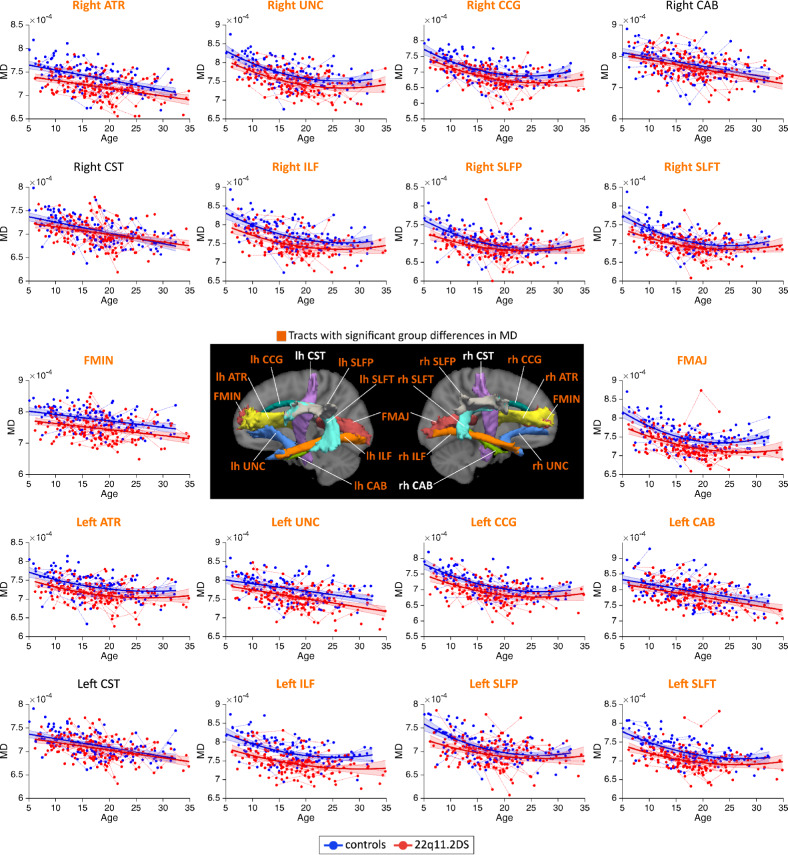
Developmental trajectories of mean diffusivity (MD) in patients with 22q11.2DS and controls. MD metrics followed linear or quadratic decreasing trajectories in 22q11.2DS (red) and controls (blue). No significant age × group interaction effects were found, indicating that patients with 22q11.2DS and controls followed parallel developmental curves with similar shapes. However, significant group differences were found in MD, with systematic reductions in individuals with 22q11.2DS for most white matter tracts. Tracts with significant group differences are highlighted in orange in plot titles and in the central figure. For a summary of all *p*-values for group and age × group interaction effects in white matter tracts, see Supplementary Materials, Table S4. The significance threshold was set at *p* < 0.05. All results were corrected for multiple comparisons using the FDR method. lh left hemisphere, rh right hemisphere, FMAJ forceps major (corpus callosum), FMIN forceps minor (corpus callosum), ATR anterior thalamic radiation, CAB cingulum–angular bundle, CCG cingulum–cingulate bundle, CST corticospinal tract, ILF inferior longitudinal fasciculus, SLFP superior longitudinal fasciculus–parietal bundle, SLFT superior longitudinal fasciculus–temporal bundle, UNC uncinate fasciculus.

**Fig. 2:**
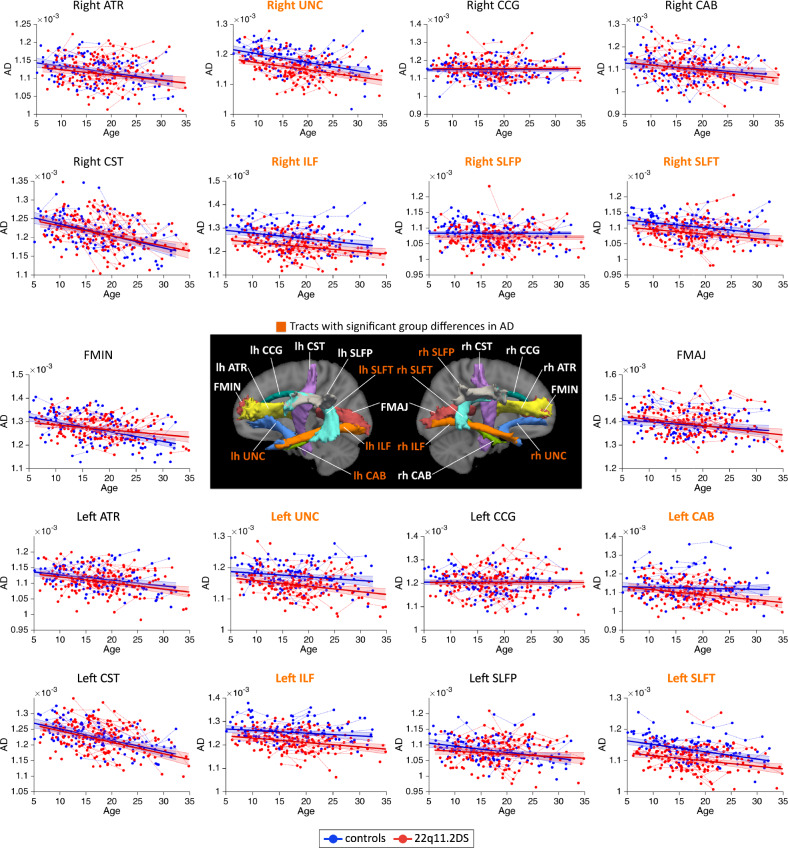
Developmental trajectories of axial diffusivity (AD) in patients with 22q11.2DS and controls. AD metrics followed linear decreasing trajectories in 22q11.2DS (red) and controls (blue) in most white matter tracts. Age × group interactions were not significant, indicating that both groups followed parallel developmental curves with similar shapes. Multiple white matter tracts showed significant group differences in AD, with systematic reductions in 22q11.2DS. Tracts with significant group differences are highlighted in orange in plot titles and in the central figure. The significance threshold was set at *p* < 0.05. All results were corrected for multiple comparisons using the FDR method. lh left hemisphere, rh right hemisphere, FMAJ forceps major, FMIN forceps minor, ATR anterior thalamic radiation, CAB cingulum–angular bundle, CCG cingulum–cingulate bundle, CST corticospinal tract, ILF inferior longitudinal fasciculus, SLFP superior longitudinal fasciculus–parietal bundle, SLFT superior longitudinal fasciculus–temporal bundle, UNC uncinate fasciculus.

**Fig. 3:**
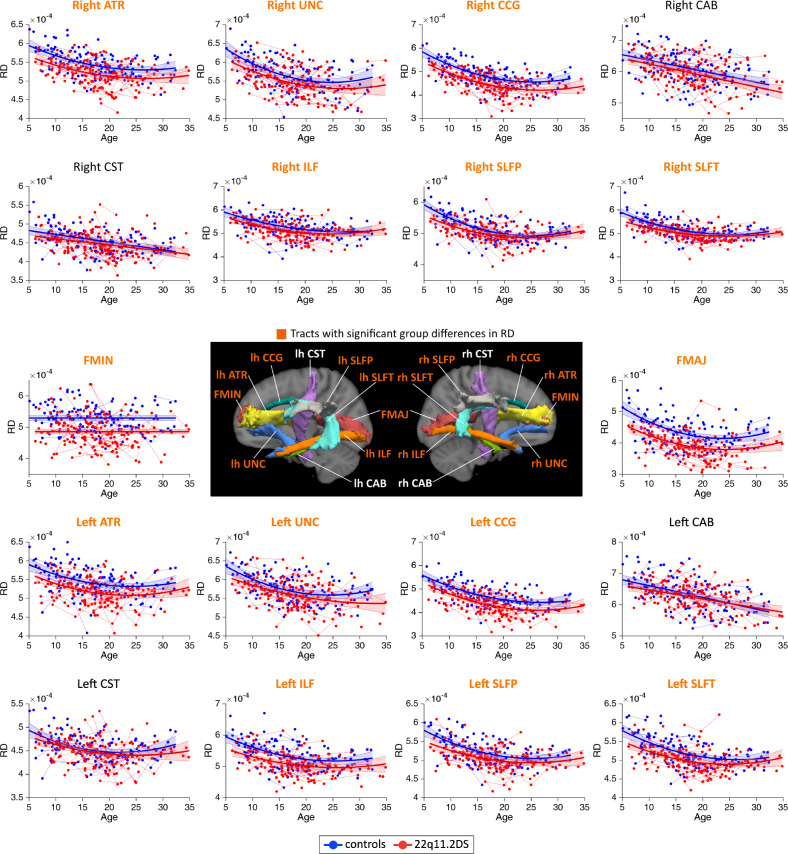
Developmental trajectories of radial diffusivity (RD) in patients with 22q11.2DS and controls. RD metrics mostly followed quadratic decreasing trajectories in 22q11.2DS (red) and controls (blue). Similar to other diffusion measures, age × group interactions were absent and both groups therefore displayed identically shaped developmental curves. Group differences were evident in a majority of white matter tracts, with systematic reductions of RD in individuals with 22q11.2DS. Tracts with significant group differences are highlighted in orange in plot titles and in the central figure. The significance threshold was set at *p* < 0.05. All results were corrected for multiple comparisons using the FDR method. lh left hemisphere, rh right hemisphere, FMAJ forceps major, FMIN forceps minor, ATR anterior thalamic radiation, CAB cingulum–angular bundle, CCG cingulum–cingulate bundle, CST corticospinal tract, ILF inferior longitudinal fasciculus, SLFP superior longitudinal fasciculus–parietal bundle, SLFT superior longitudinal fasciculus–temporal bundle, UNC uncinate fasciculus.

**Fig. 4:**
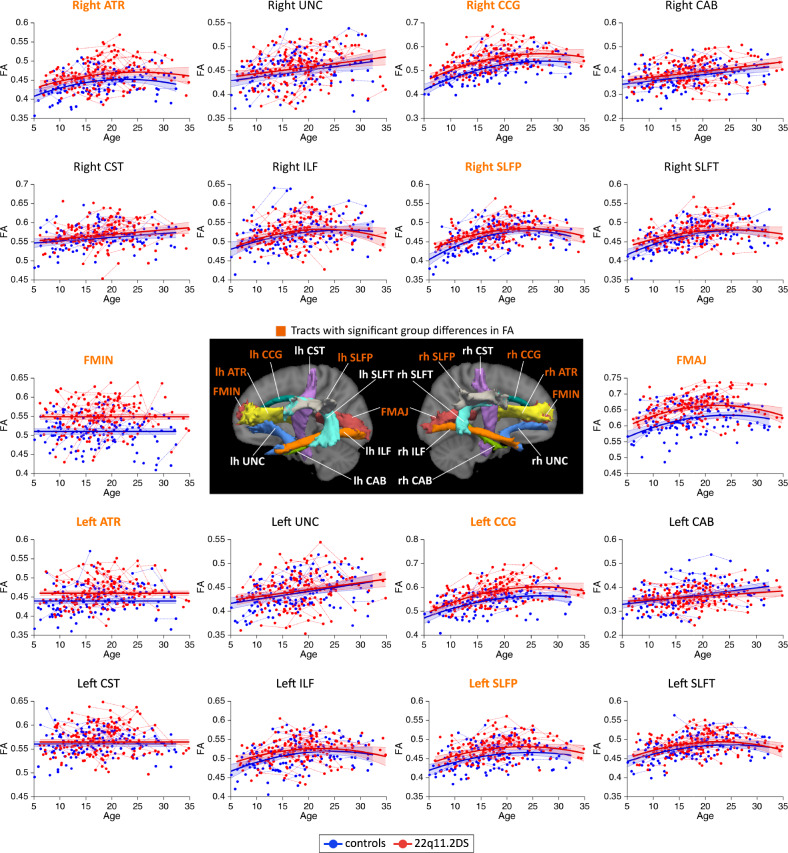
Developmental trajectories of fractional anisotropy (FA) in patients with 22q11.2DS and controls. FA metrics followed increasing quadratic or linear developmental curves in 22q11.2DS (red) and controls (blue). Age × group interaction effects were again not significant, revealing parallel trajectories in both groups. Group differences were visible in a number of tracts, where FA was systematically increased in 22q11.2DS. Tracts with significant group differences are highlighted in orange in plot titles and in the central figure. The significance threshold was set at *p* < 0.05. All results were corrected for multiple comparisons using the FDR method. lh left hemisphere, rh right hemisphere, FMAJ forceps major, FMIN forceps minor, ATR anterior thalamic radiation, CAB cingulum–angular bundle, CCG cingulum–cingulate bundle, CST corticospinal tract, ILF inferior longitudinal fasciculus, SLFP superior longitudinal fasciculus–parietal bundle, SLFT superior longitudinal fasciculus–temporal bundle, UNC uncinate fasciculus.

MD measures followed linear or quadratic decreasing trajectories in 22q11.2DS and controls (Fig. 1). Patients with 22q11.2DS showed significant reductions in MD for a majority of white matter tracts. AD metrics followed linear decreasing trajectories in both groups and most white matter tracts (Fig. 2). Similar to MD, AD metrics showed significant reductions in 22q11.2DS, although these differences were present in fewer tracts. RD metrics underwent decreasing trajectories in 22q11.2DS and controls, with a majority of tracts showing quadratic developmental curves (Fig. 3). Significant group differences were evident in most white matter tracts, with a reduction of RD in individuals with 22q11.2DS. Finally, FA metrics followed increasing quadratic or linear developmental curves in a majority of tracts (Fig. 4). Patients with 22q11.2DS showed significantly increased FA values in several tracts.

### PLS correlation between white matter microstructure and clinical risk factors of psychosis

**Fig. 5:**
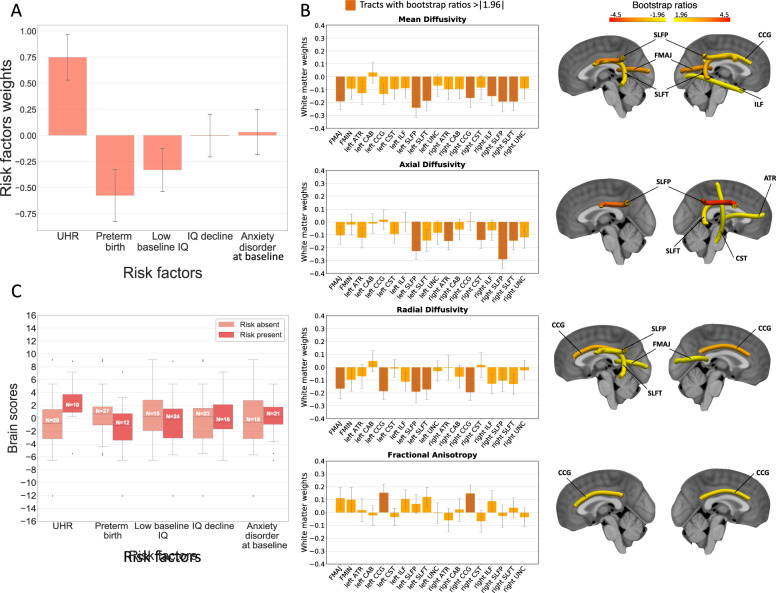
Relationship between clinical risk factors of psychosis and white matter microstructure in 22q11.2DS captured through PLS correlation. PLS correlation analysis between white matter microstructure and clinical risk factors of psychosis in subjects with 22q11.2DS revealed one significant correlation component (*r* = 0.48, *p* = 0.027), as evaluated by permutation testing (threshold of *p* < 0.05). **A** Corresponding behavior weights of risk factors are displayed with bootstrap error bars; **B** brain white matter weights are represented with bootstrap error bars. Tracts that most heavily contribute to the brain-behavior correlation, that is, with bootstrap ratios >1.96 or <−1.96 (corresponding to a robustness at a confidence interval of 95%) are highlighted in darker orange in the graphs and displayed in the brain renderings, where tracts with orange to red colors contribute most strongly to the brain-behavior correlation; **C** finally, brain scores are plotted for groups of patients carrying (risk present) or not (risk absent) a given risk factor. The correlation pattern reveals that the UHR status, preterm birth, and low baseline IQ strongly contribute to the brain-behavior pattern. Interestingly, patients at UHR present opposite tendencies of brain alterations (reduced AD, RD, MD, and increased FA) compared to subjects born preterm or with a low IQ at baseline (increased AD, RD, MD, and decreased FA). White matter tracts furthermore present consistent and widespread alterations affecting multiple tracts and all four diffusion metrics. lh left hemisphere, rh right hemisphere, FMAJ forceps major (corpus callosum), FMIN forceps minor (corpus callosum), ATR anterior thalamic radiation, CAB cingulum–angular bundle, CCG cingulum–cingulate bundle, CST corticospinal tract, ILF inferior longitudinal fasciculus, SLFP superior longitudinal fasciculus–parietal bundle, SLFT superior longitudinal fasciculus–temporal bundle, UNC uncinate fasciculus.

PLS correlation between white matter microstructure and risk factors of psychosis within 22q11.2DS (*N* = 39) revealed one significant correlation component (*r* = 0.48, *p* = 0.027). Corresponding brain (white matter microstructure) and behavior (clinical risk factors) weights are displayed in Fig. 5. Behavior weights indicated a strong contribution of UHR (risk factor weight 0.75 ± 0.21), preterm birth (risk factor weight −0.57 ± 0.23), and low baseline IQ (risk factor weight −0.32 ± 0.19) to the observed brain-behavior correlation. By contrast, risk factors such as cognitive decline (risk factor weight −0.02 ± 0.21) or anxiety disorder at baseline (risk factor weight 0.03 ± 0.20) only weakly impacted the correlation. Interestingly, brain scores of the UHR group were higher than the non-UHR group, indicating that individuals at UHR showed neuroanatomical characteristics that were similar to the brain pattern displayed in Fig. 5B, involving a pattern of reductions in AD, RD, and MD combined with increased FA. Of note, reductions here reflect lower diffusion measures with respect to the average developmental curve in 22q11.2DS at that age. By contrast, patients presenting risk factors such as low baseline IQ or preterm birth showed lower brain scores, meaning that they were characterized by very different, opposite neuroanatomical features involving increased AD, RD, and MD combined with decreased FA. The association between brain and behavior scores of the significant correlation component is displayed in Supplementary Figure S4.

## Discussion

In this study, we found that 22q11.2DS and controls showed parallel developmental trajectories of white matter microstructure with similar shapes in all tracts and diffusion metrics, as indicated by the absence of age × group interaction effects. Group differences of white matter development were however particularly present and were characterized by consistently decreased MD, AD, RD, and increased FA in individuals with 22q11.2DS compared to controls, providing evidence for structural dysconnectivity in individuals at genetic high risk for psychosis. Our multivariate PLS analysis furthermore revealed distinctive patterns of white matter alterations in individuals with 22q11.2DS presenting clinical risk factors of psychosis, where UHR was associated with a pattern of decreased MD, AD, RD, and increased FA, and conversely, low baseline IQ and prematurity were associated with an opposite pattern of increased MD, AD, RD, and decreased FA.

In agreement with tractography studies of normative brain white matter development^24–26,61^, we found mostly constant or linearly decreasing trajectories in AD, progressive non-linear increasing trajectories in FA, and non-linear decreasing trajectories in RD and MD with continued maturation during adulthood, particularly in association tracts. However, contrary to previous cross-sectional findings which suggested a lack or delayed white matter maturation in 22q11.2DS^20–22^, longitudinal modeling revealed trajectory shapes in 22q11.2DS that were similar to controls, indicating a preservation of age-related changes in the syndrome.

Moreover, in line with previous DTI studies in 22q11.2DS, current evidence confirms the widespread nature of white matter alterations in the syndrome^19^. While the direction (increases vs decreases) of observed anomalies was inconsistent in previous smaller sample studies^19^, a recent multisite cross-sectional study provided a clearer pattern of decreased AD, RD, and MD combined with a mixed pattern of increased and decreased FA depending on the tracts^22^. Using the largest longitudinal DTI sample analyzed to date, our study largely confirmed these findings, as we found widespread and highly consistent reductions in AD, RD, and MD, and increased FA affecting most white matter tracts. Of note, we did not find evidence for FA reductions in 22q11.2DS. It is plausible that longitudinal analyses were optimally suited to capture complex developmental trajectories, resulting in a more consistent pattern of alterations. Alternatively, this difference may also be related to methodological differences or variations in included age ranges.

On a neurobiological level, murine models suggest that reductions in RD are indicative of excessive myelination of axonal tracts^62^, whereas reductions in AD may be driven by reduced tract organization, axonal disruption, or a reduction in the diameter of axonal tracts^62–65^. While the biological meaning of FA is still debated, increases in this metric have been suggested to reflect reduced axonal branching, which would in turn lead to a reduced amount of fiber crossings^66^. Interestingly, a recent study using a novel multi-tensor anisotropy measure provided further evidence in support of this hypothesis, as they found similarly increased FA in regions with major fiber crossings in 22q11.2DS^67^. Other speculations regarding the cellular nature of increased FA include flattened bundles allowing for increased white matter density^68^, or fewer arched fibers^69^. Importantly, however, murine models of white matter pathology show an interdependence of FA and RD^64^, as white matter bundles become more anisotropic when myelination is increased (i.e., RD is reduced). Accordingly, our findings suggest that 22q11.2DS is characterized by a combination of excessive myelination processes and disrupted tract organization, where fiber bundles may be composed of thinner, less numerous, or more loosely packed axons. Of note, disrupted myelination has been critically related to impaired Glutamatergic-GABAergic neurotransmission leading to brain circuit asynchrony, a highly consistent finding in schizophrenia^4^ that has been recently confirmed in 22q11.2DS^70^. Furthermore, it is thought that myelination is partially influenced by neuronal activity^71^. It is possible that aberrant neurotransmission and white matter microstructure mutually influence each other throughout development to generate brain asynchrony. While the exact relationship between these mechanisms still remains to be determined, our findings add evidence suggesting that a strong genetic risk for psychosis induces profound structural alterations that likely increase the vulnerability for psychosis, potentially by participating in disruptions of brain synchrony.

Using a multivariate PLS correlation analysis, this study furthermore sought to identify the neural correlates of clinical risk factors of psychosis in 22q11.2DS. Multivariate modeling revealed that UHR was a strongly discriminating factor associated with a very distinctive neuroanatomical pattern of white matter alterations, characterized by consistently increased FA and reduced AD, RD, and MD. Other strongly contributing risk factors were preterm birth and low baseline IQ, but interestingly, these presented an opposite pattern of brain alterations involving increased AD, RD, MD, and reduced FA. While this finding was unexpected, we can speculate that the psychosis risk conveyed by the UHR risk factor involves distinct neurobiological mechanisms that are unrelated to structural alterations at play for other risk factors. The exact neurobiological underpinnings related to these clinical features are, however, yet to be uncovered. Our results also revealed that anxiety disorders at baseline and IQ decline were only weakly associated with the brain pattern, suggesting that white matter alterations are distinctively associated with some specific clinical features of high risk for transition. Overall, shifting to a multivariate approach provided a way to quantify the specific relationship of multiple clinical measures with neuroanatomical structure and revealed for the first time a differential involvement of white matter pathology in different clinical risk factors of psychosis, thereby supporting the existence of multiple neuropathological pathways to the disease.

Importantly, multivariate modeling revealed very consistent and widespread patterns of brain alterations, in line with recent evidence indicating that white matter alterations of first-episode and chronic stages of schizophrenia are very diffuse and affect the brain on a global level^9,38,39^. While studies have also demonstrated an involvement of white matter alterations in preclinical high-risk stages of psychosis in the general population^8^ and in patients with 22q11.2DS presenting psychotic symptoms^19,34–36^ or a cognitive decline^37^, results were heterogeneous and prevented the identification of a reliable biomarker, likely because of the variability and small magnitude of premorbid anomalies. Current evidence suggests that, on the other hand, a multivariate assessment of brain structure can effectively identify early, more subtle patterns of alterations. This evidence therefore supports an increasingly dominant trend in schizophrenia research, arguing that pattern-based biomarkers identified using multivariate methods offer very promising clinical applications and may greatly improve predictive accuracy^72^, as they can capture alterations that were likely too subtle or too variable to make sense of using univariate techniques.

Interestingly, the delineation of white matter developmental trajectories in 22q11.2DS and multivariate assessment of clinical risk factors may provide critical information regarding the timing of neurodevelopmental events associated with strong genetic and clinical vulnerability for psychosis. Specifically, the presence of widespread white matter alterations combined with normative-like developmental trajectories in 22q11.2DS suggests that syndrome-related anomalies of white matter microstructure appear very early on, during prenatal or early childhood developmental stages. Considering the major role of genetic programming during brain formation in utero and infancy, it is plausible that the haploinsufficiency caused by the 22q11.2 deletion acts like an “early hit” which already strongly increases the vulnerability for psychosis^4,73^. Additional insults occurring during early brain development, such as preterm birth, can further increase this vulnerability, as shown by our multivariate analysis. On the other hand, the association between specific brain alterations and risk factors emerging during late childhood and adolescence (UHR, low baseline IQ) suggests that vulnerability for schizophrenia may also involve a “late” neurodevelopmental hit, affecting brain reorganization and white matter maturation processes occurring at subsequent developmental stages. Taken together, our results support the “two-hit” theory of schizophrenia, suggesting that the disorder results from several waves of detrimental developmental events^73^.

The current findings highlight several important translational research directions. First, white matter microstructure should be assessed in animal models of 22q11.2DS using MRI to confirm the present results, as white matter microstructure of such models has not yet been fully characterized (for reviews of the current understanding of the neurobiology underlying 22q11.2DS, see refs. ^14,74^). Next, cellular and molecular investigations of pre-and perinatal phases are needed to determine the exact underlying mechanisms and timing of developmental events that drive white matter alterations observed in this study. Particularly, the hypothesis of reduced fiber crossings in 22q11.2DS, as well as the potential presence of excessive myelination are promising areas of investigation. Of interest, several genes within the 22q11.2 locus are involved in myelin-related signaling pathways (e.g., RTN4R^75^, PIK4CA^76^) or axonal growth and branching (ZDHHC8^77^) and may therefore play a key role in the emergence of white matter disruptions. Finally, as some risk factors such as cognitive deficits can be readily measured in mice, it will be important to confirm their association with the emergence of additional white matter disruptions during development. Collectively, these lines of work may provide a neurobiological confirmation of the two-hit theory of schizophrenia and may further delineate mechanisms underlying white matter dysconnectivity in the illness.

This study has some limitations. First, while this is the first study delineating developmental trajectories of white matter microstructure using longitudinal data with up to three-time points, larger longitudinal samples including a higher number of time points per subject and a longer total follow-up time will be needed to further improve the characterization of white matter maturation. Second, significant differences in IQ were observed between patients with 22q11.2DS and controls. While lower IQ is an inherent characteristic of the syndrome that is difficult to disentangle from its other phenotypic features, future studies including IQ-matched controls are warranted to fully address the potential role of IQ in white matter alterations. A third limitation concerns the limited sample size available for the multivariate PLS analysis of risk factors for psychosis (*N* = 39), implying that these results should be considered as preliminary and will need confirmation in larger samples. Relatedly, larger samples may provide an opportunity for outcome prediction studies, which are strongly needed to further consolidate early detection and intervention methods. Fourth, the DTI model of diffusion may be suboptimal in assessing complex brain regions with a high amount of fiber crossings, as it forces the estimation of a single average direction of diffusion in each voxel^78,79^. Studies using more complex, high-resolution image acquisitions for white matter microstructure are currently underway. Despite this limitation, the DTI model is widely used and accepted as a valid model for assessing white matter microstructure in humans. Finally, scans were acquired using two different MRI scanners and head coils (Siemens Trio with a 12 channels head coil; Siemens Prisma with a 20 channels head coil). The proportion of scans was however similar in 22q11.2DS and controls as well as in all risk factor groups (see Supplementary Material, Table S1), and this factor was included as a covariate in our analyses to account for its potentially confounding effect.

To conclude, findings from this study provide strong evidence for precocious neurodevelopmental anomalies of white matter structure in 22q11.2DS, clarifying the role of genetic high risk for psychosis in white matter maturation. Results furthermore indicate that additional clinical risk factors for psychosis further impact white matter development, with a differential involvement of UHR, preterm birth, and low IQ at baseline. As such, longitudinal and multivariate approaches of white matter microstructure represent effective means to capture complex developmental brain alterations preceding psychosis and show potential for the identification of predictive and prognostic biomarkers. Future studies using larger samples should be conducted to further confirm the neurodevelopmental underpinnings of genetic and clinical risk factors of premorbid psychosis. A particular emphasis should be placed on the collection of samples that include both the preclinical stage, as well as the transition to clinical stages of psychosis. An overview of the full trajectory to psychosis will indeed provide means to assess the predictive accuracy of alterations identified in preclinical stages, which will be a critical step to further improve our understanding of the neuropathological pathways leading to psychosis.

## Supplementary information

Supplemental Material
