# Supplementary material for: Identifying neurodevelopmental anomalies of white matter microstructure associated with high risk for psychosis in 22q11.2DS
Source: Transl Psychiatry. 2020 Nov 24;10:408. doi: 10.1038/s41398-020-01090-z (PMC7686319; doi:10.1038/s41398-020-01090-z)
Supplement: Supplementary file 1 — Supplemental Material [file 41398_2020_1090_MOESM1_ESM.docx]

**Supplementary Materials for: Identifying neurodevelopmental anomalies of white matter microstructure associated with high risk for psychosis in 22q11.2DS**

Joëlle Bagautdinova^1^, Maria C. Padula^1^, Daniela Zöller^1,2,3,4^, Corrado Sandini^1^, Maude Schneider^1,5^, Marie Schaer^1^, Stephan Eliez^1^

^1^ Developmental Imaging and Psychopathology Laboratory, University of Geneva School of Medicine, Geneva, Switzerland

^2^ Medical Image Processing Laboratory, Institute of Bioengineering, École Polytechnique Fédérale de Lausanne (EPFL), Lausanne, Switzerland

^3^ Department of Radiology and Medical Informatics, University of Geneva, Geneva, Switzerland

^4^ Institute of Neuromodulation and Neurotechnology, Department of Neurosurgery and Neurotechnology, University of Tübingen, Germany

^5^ Clinical Psychology Unit for Intellectual and Developmental Disabilities, Faculty of Psychology and Educational Sciences, University of Geneva, Geneva, Switzerland

[Supplementary Method 3](#_Toc53157342)

[Image quality check 3](#_Toc53157343)

[MRI processing 3](#_Toc53157344)

[Head motion 4](#_Toc53157345)

[Supplementary Figures 5](#_Toc53157346)

[Supplementary Figure S1. 5](#_Toc53157347)

[Supplementary Figure S2. 6](#_Toc53157348)

[Supplementary Figure S3. 7](#_Toc53157349)

[Supplementary Figure S4. 9](#_Toc53157350)

[Supplementary Tables 10](#_Toc53157351)

[Supplementary Table S1. 10](#_Toc53157352)

[Supplementary Table S2. 11](#_Toc53157353)

[Supplementary Table S3. 12](#_Toc53157354)

[Supplementary Table S4. 13](#_Toc53157355)

[References 15](#_Toc53157356)

## Supplementary Method

#### Image quality check

T1-weighted and DTI scans were visually inspected by two trained raters (first and second authors, JB and MP). Scans with excessive head movement, major artefacts or where parts of the cortex were not fully captured (63 scans, comprising 46 22q11.2DS scans and 17 controls scans) were excluded prior to sample selection. The selected sample for the analyses of developmental trajectories in 22q11.2DS and controls thus comprised 302 quality-checked scans.

#### MRI processing

First, anatomical segmentation and parcellation of T1-weighted structural scans was performed using the longitudinal pipeline of FreeSurfer v6.0 (for details on processing steps see <http://surfer.nmr.mgh.harvard.edu/>), where all scans of a given individual are used to create an unbiased within-subject template^1^. Common information of the within-subject template is then taken into account during the processing of scans from each time point. This method has been shown to increase reliability and statistical power, thereby improving the estimation of within-subject age-related changes^2^. Scans and within-subject templates were manually reviewed and corrected where necessary.

Next, diffusion weighted images were processed using the longitudinal pipeline of TRActs Constrained by UnderLying Anatomy (TRACULA, v6.0)^3^, an automated global probabilistic tractography algorithm provided by FreeSurfer. Preprocessing involves eddy current distortion correction, reorientation of gradient vectors, intra-subject registration (alignment of DW images to the T1-weighted scan from the same time point using Freesurfer’s bbregister function), inter-subject registration (alignment to MNI152 template^4^), brain mask extraction using the T1-weighted anatomical information, computation of head motion measures and fitting of a diffusion tensor model using FSL’s dtifit (<http://www.fmrib.ox.ac.uk/fsl>) to generate the fractional anisotropy (FA), axial diffusivity (AD), radial diffusivity (RD) and mean diffusivity (MD) measures. Of note, the tensor fit is only performed to extract tensor-based measures for later analyses and is not used to perform tractography; instead, TRACULA uses FSL’s bedpostX to apply a more complex “ball-and-stick” diffusion model.

The longitudinal stream of TRACULA then estimates the probability distribution of 18 major white matter tracts given the T1-weighted and DW information from all available time points. The probability distribution of a pathway is computed partly using the “ball-and-stick” model of diffusion mentioned above, and partly using prior anatomical information about white matter tracts based on a set of training subjects in whom tracts have been labelled manually. More specifically, the prior information used for tractography is the likelihood of a pathway to traverse (or pass next to) anatomical segmentation labels. This procedure enables efficient tracts reconstruction even in the presence of tracts shape or size differences among analyzed scans. Moreover, TRACULA performs tract reconstruction in the native space of the subject to ensure that the same white matter parts are compared between time points. This has been demonstrated to improve test-retest reliability and increase sensitivity to longitudinal changes in white matter tracts^3^, making it a particularly adapted tool for longitudinal studies of white matter development. Once the tracts distributions have been estimated, TRACULA extracts four diffusion measures per tract: FA, AD, RD and MD. Diffusion metrics are provided as averages over each tract. Tracts reconstruction was manually verified in each individual scan and was unsuccessful in three subjects (two patients with 22q11.2DS, one control), resulting in a sample of 199 subjects for the characterization of white matter development.

#### Head motion

We verified the four head motion scores provided after tracts reconstruction using TRACULA (for detailed explanations regarding the computation of the motion scores, see^5^), which are average translation (mm), average rotation (degrees), percent of bad slices (%) and average dropout score. None of the motion measures had significant differences between 22q11.2DS and controls, indicating that head motion does not represent a major confounding factor in the analyses. Table S2 contains a description of mean and standard deviation for all four motion parameters outputted by TRACULA, as well as p-values of group comparisons of each motion measure.

##

## Supplementary Figures

Supplementary Figure S1. Age distributions of participants included in the study. Panel A shows the age distribution of participants included in the first analysis comparing white matter development in 22q11.2DS (N=101) and controls (N=100) using mixed models regression. Among these participants, one hundred twenty subjects had a single visit (N=52 22q11.2DS, N=68 controls), 61 subjects had two visits (N=33 22q11.2DS; N=28 controls) and 20 subjects had three visits (N=16 22q11.2DS; N=4 controls), resulting in a total of 302 scans. Panel B displays the age distribution of participants included in the second analysis assessing the impact of clinical risk factors on white matter development in 22q11.2DS (N=39) using multivariate PLS correlation. The sample included 29 participants with two visits and 10 participants with 3 visits, resulting in a total of 88 scans.

Supplementary Figure S2. Illustration of the 18 white matter tracts reconstructed by TRACULA**.**

Supplementary Figure S3. Scheme displaying the different steps of the Partial Least Squares (PLS) correlation analysis. First, to create the brain matrix (**X**), mixed models computing the relationship between age and each brain measure in the group of all 39 patients with 22q11.2DS were estimated. As there are 18 tracts x 4 diffusion metrics, 72 models were fitted. We then computed the average diffusion metric for each white matter tract across the scans of each subject, resulting in 72 diffusion metrics per subject (one average measure for 18 tracts x 4 diffusion metrics). Finally, in order to account for age, we extracted the residuals (i.e., the difference between the observed and predicted values of the models fitted in the first step) for all subjects in each of the 72 measures. These residuals can be considered as a summary measure indicating the deviation of a given subject with respect to the predicted development at corresponding ages and were used as brain measures. Thus, the resulting brain matrix (**X**) was a 39 (subjects) by 72 (white matter measures) matrix. Five dichotomized risk factors (UHR, baseline FSIQ, cognitive decline, preterm birth and anxiety disorder at baseline) were entered in the matrix (**Y**), resulting in a 39 (subjects) by 5 (risk factors) matrix. Then, **X** and **Y** were z-scored across subjects, and the correlation matrix **R** was computed trough **R = Y^T^X**, resulting in a 5 (risk factors) by 72 (white matter measures) matrix containing the correlation between each risk factor and each white matter measure across subjects. The main correlation components were then extracted through singular value decomposition (SVD) of the correlation matrix **R = USV**. As R was a 5x72 matrix, a total of five components was extracted. For each correlation component, the singular value (on the diagonal of **S**) reveals the amount of correlation explained by the component, while the behavior weights (columns of **U**) and brain weights (rows of **V**) indicate how strongly the original behavior and brain variables respectively contribute to the brain-behavior correlation.

Supplementary Figure S4. Correlation between individual brain (**L_x_**) and behavioral scores (**L_y_**) of participants (r=.48). Brain and behavior scores are obtained by projecting the original brain (**X**) and behavior (**Y**) matrices into their respective brain (**V**) and behavior (**U**) weights, and indicate each individual’s brain and behavior contribution to the significant correlation component.


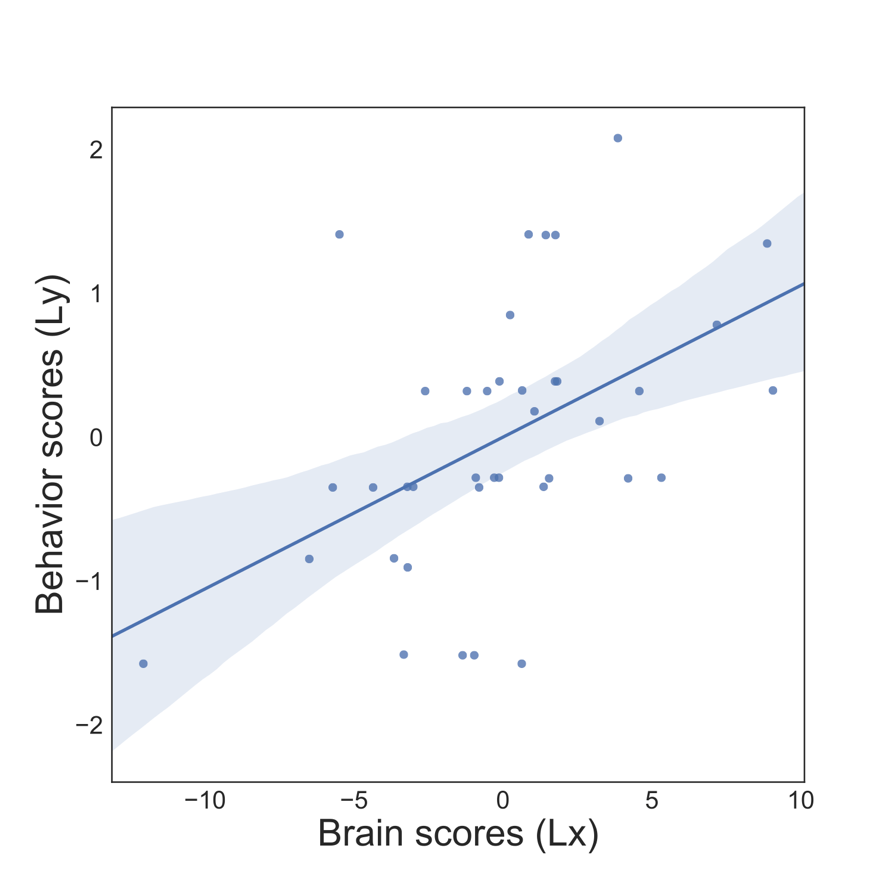


## Supplementary Tables

Supplementary Table S1. Demographic information of participants included in the study.

|  | **22q11.2DS** | **controls** | **Total** | **p-value** |
| --- | --- | --- | --- | --- |
| Number of participants included in study | 101 | 100 | 201 |  |
| Participants with 1 visit | 52 | 68 | 120 |  |
| Participants with 2 visits | 33 | 28 | 61 |  |
| Participants with 3 visits | 16 | 4 | 20 |  |
| Total number of visits | 166 | 136 | 302 |  |
| Proportion of males/females | 51/50 | 48/52 | 99/102 | 0.724 |
| Mean age at first visit (SD) | 15.952 (6.037) | 16.684 (6.797) |  | 0.421 |
| Mean FSIQ at first visit (SD) | 70.71 (11.489) | 111.58 (14.436) |  | < 0.001 |
| Mean time interval between visits (SD) | 3.673 (0.893) | 3.338 (0.784) |  | 0.054 |
| Number of participants with psychiatric diagnosis at first visit | 65 |  |  |  |
| Attention deficit disorder | 26 |  |  |  |
| Anxiety disorder | 50 |  |  |  |
| Mood disorder | 17 |  |  |  |
| Psychotic disorder | 6 |  |  |  |
| Schizophrenia | 2 |  |  |  |
| More than one psychiatric disorder | 44 |  |  |  |
| Number of participants medicated at first visit | 31 |  |  |  |
| Methylphenidate | 13 |  |  |  |
| Antidepressants | 8 |  |  |  |
| Antipsychotics | 6 |  |  |  |
| Anticonvulsants | 6 |  |  |  |
| Anxiolytics | 4 |  |  |  |
| More than one type of medication | 6 |  |  |  |

Supplementary Table S2. Proportion of scans acquired using each of the two scanners and respective head coils (3T Siemens Trio with 12 channels head coil; 3T Siemens Prisma with 20 channels head coil). There was no significant difference of scanner (and respective head coil) distribution between diagnostic groups *X*^2^(1, *N* = 302) = 0.233, *p* = 0.629) or between any of the risk factor groups within 22q11.2DS, including the Ultra-High-Risk factor *X*^2^(1, *N* = 88) = 0.374, *p* = 0.541), baseline IQ *X*^2^(1, *N* = 88) = 0.001, *p* = 0.975), IQ decline *X*^2^(1, *N* = 88) = 0.624, *p* = 0.429), preterm birth *X*^2^(1, *N* = 88) = 0.844, *p* = 0.358) and anxiety disorder at baseline *X*^2^(1, *N* = 88) = 0.024, *p* = 0.878), indicating that scanner and head coils may not have confounded results.

|  |  | **Scanner & head coil** | |
| --- | --- | --- | --- |
|  |  | 3T Siemens Trio,  12 ch. head coil | 3T Siemens Prisma,  20 ch. head coil |
| **Diagnosis** | controls | 84 | 52 |
|  | 22q11.2DS | 107 | 59 |
| **UHR** | UHR | 15 | 6 |
|  | non-UHR | 43 | 24 |
| **Baseline IQ** | baseline IQ < 75 | 35 | 18 |
|  | baseline IQ ≥ 75 | 23 | 12 |
| **IQ decline** | IQ decline | 22 | 14 |
|  | no IQ decline | 36 | 16 |
| **Preterm birth** | born preterm | 19 | 7 |
|  | born at term | 39 | 23 |
| **Anxiety disorder at baseline** | anxiety disorder at baseline | 30 | 15 |
|  | no anxiety disorder at baseline | 28 | 15 |

Supplementary Table S3. Mean and standard deviation of motion parameters provided by TRACULA. None of the motion measures differed significantly between 22q11.2DS and controls.

|  | **Diagnosis** | | |
| --- | --- | --- | --- |
|  | Controls | 22q11.2DS | **p-value** |
| **Average translation (mm)** | 0.795 (0.231) | 0.833 (0.27) | 0.195 |
| **Average rotation (degrees)** | 0.005 (0.002) | 0.006 (0.002) | 0.684 |
| **Percent of bad slices (%)** | 0.009 (0.068) | 0.004 (0.021) | 0.401 |
| **Average dropout score** | 1.013 (0.062) | 1.005 (0.036) | 0.223 |

Supplementary Table S4. Mixed models comparing Axial Diffusivity (AD), Radial Diffusivity (RD), Mean Diffusivity (MD) and Fractional Anisotropy (FA) development in patients with 22q11.2DS and controls. For each diffusion measure and tract, the type of fitted model (constant, linear or quadratic) and p-values for group and age x group interaction effects are reported. Significant effects are indicated in bold. All results were corrected for multiple comparisons using the FDR method.

Legends: FMAJ - forceps major (corpus callosum); FMIN - forceps minor (corpus callosum); ATR - anterior thalamic radiation; CAB - cingulum, angular bundle; CCG - cingulum, cingulate bundle; CST - corticospinal tract; ILF - inferior longitudinal fasciculus; SLFP - superior longitudinal fasciculus, parietal bundle; SLFT - superior longitudinal fasciculus, temporal bundle; UNC - uncinate fasciculus.

|  |  | |  |  | **MD** |  |  |  | **AD** |  |  |
| --- | --- | --- | --- | --- | --- | --- | --- | --- | --- | --- | --- |
| **Tract type** | **Hemisphere** | **Tract** | |  | **Model** | **Group effect** | **Interaction effect** |  | **Model** | **Group effect** | **Interaction effect** |
| Projection | left | ATR | |  | Quadratic | **< 0.001** | 1 |  | Linear | 0.352 | 0.898 |
|  | right | ATR | |  | Linear | **< 0.001** | 0.623 |  | Linear | 0.411 | 0.762 |
|  | left | CST | |  | Linear | 0.052 | 0.88 |  | Linear | 0.558 | 0.958 |
|  | right | CST | |  | Linear | 0.076 | 0.591 |  | Linear | 0.837 | 0.762 |
| Commissural |  | FMAJ | |  | Quadratic | **< 0.001** | 0.623 |  | Linear | 0.558 | 0.635 |
|  |  | FMIN | |  | Linear | **< 0.001** | 0.981 |  | Linear | 0.103 | 0.074 |
| Association | left | CAB | |  | Linear | **0.009** | 1 |  | Linear | **0.001** | 0.14 |
|  | right | CAB | |  | Linear | 0.146 | 0.981 |  | Linear | 0.837 | 0.762 |
|  | left | CCG | |  | Quadratic | **< 0.001** | 0.88 |  | Constant | 0.86 | n.a. |
|  | right | CCG | |  | Quadratic | **< 0.001** | 0.981 |  | Constant | 0.411 | n.a. |
|  | left | ILF | |  | Quadratic | **< 0.001** | 0.933 |  | Linear | **< 0.001** | 0.711 |
|  | right | ILF | |  | Quadratic | **< 0.001** | 0.88 |  | Linear | **< 0.001** | 0.774 |
|  | left | SLFP | |  | Quadratic | **< 0.001** | 0.591 |  | Linear | 0.15 | 0.457 |
|  | right | SLFP | |  | Quadratic | **< 0.001** | 0.591 |  | Constant | **0.007** | n.a. |
|  | left | SLFT | |  | Quadratic | **< 0.001** | 0.623 |  | Linear | **< 0.001** | 0.635 |
|  | right | SLFT | |  | Quadratic | **< 0.001** | 0.591 |  | Linear | **< 0.001** | 0.898 |
|  | left | UNC | |  | Linear | **< 0.001** | 0.88 |  | Linear | **< 0.001** | 0.711 |
|  | right | UNC | |  | Quadratic | **< 0.001** | 0.981 |  | Linear | **< 0.001** | 0.762 |

|  |  |  |  | **RD** |  |  |  | **FA** |  |  |
| --- | --- | --- | --- | --- | --- | --- | --- | --- | --- | --- |
| **Tract type** | **Hemisphere** | **Tract** |  | **Model** | **Group effect** | **Interaction effect** |  | **Model** | **Group effect** | **Interaction effect** |
| Projection | left | ATR |  | Quadratic | **< 0.001** | 0.975 |  | Constant | **< 0.001** | n.a. |
|  | right | ATR |  | Quadratic | **< 0.001** | 0.975 |  | Quadratic | **0.001** | 0.966 |
|  | left | CST |  | Quadratic | 0.178 | 0.963 |  | Constant | 0.289 | n.a. |
|  | right | CST |  | Linear | 0.074 | 0.963 |  | Linear | 0.258 | 0.966 |
| Commissural |  | FMAJ |  | Quadratic | **< 0.001** | 0.963 |  | Quadratic | **< 0.001** | 0.966 |
|  |  | FMIN |  | Constant | **< 0.001** | n.a. |  | Constant | **< 0.001** | n.a. |
| Association | left | CAB |  | Linear | 0.193 | 0.963 |  | Linear | 0.258 | 0.428 |
|  | right | CAB |  | Linear | 0.098 | 0.975 |  | Linear | 0.122 | 0.966 |
|  | left | CCG |  | Quadratic | **< 0.001** | 0.975 |  | Quadratic | **< 0.001** | 0.966 |
|  | right | CCG |  | Quadratic | **< 0.001** | 0.975 |  | Quadratic | **< 0.001** | 0.966 |
|  | left | ILF |  | Quadratic | **< 0.001** | 0.963 |  | Quadratic | 0.09 | 0.966 |
|  | right | ILF |  | Quadratic | **0.008** | 0.963 |  | Quadratic | 0.605 | 0.966 |
|  | left | SLFP |  | Quadratic | **< 0.001** | 0.963 |  | Quadratic | **0.001** | 0.966 |
|  | right | SLFP |  | Quadratic | **0.001** | 0.728 |  | Quadratic | **0.023** | 0.962 |
|  | left | SLFT |  | Quadratic | **< 0.001** | 0.963 |  | Quadratic | 0.108 | 0.966 |
|  | right | SLFT |  | Quadratic | **< 0.001** | 0.728 |  | Quadratic | 0.108 | 0.734 |
|  | left | UNC |  | Quadratic | **0.006** | 0.963 |  | Linear | 0.605 | 0.966 |
|  | right | UNC |  | Quadratic | **0.005** | 0.963 |  | Linear | 0.351 | 0.966 |

## References

1. Reuter, M. & Fischl, B. Avoiding asymmetry-induced bias in longitudinal image processing. *NeuroImage* **57**, 19–21 (2011).

2. Reuter, M., Schmansky, N. J., Rosas, H. D. & Fischl, B. Within-subject template estimation for unbiased longitudinal image analysis. *NeuroImage* **61**, 1402–1418 (2012).

3. Yendiki, A., Reuter, M., Wilkens, P., Rosas, H. D. & Fischl, B. Joint reconstruction of white-matter pathways from longitudinal diffusion MRI data with anatomical priors. *NeuroImage* **127**, 277–286 (2016).

4. Talairach, J. & Tournoux, P. Co-planar stereotaxic atlas of the human brain. 1988. *Theime Stuttg. Ger* **270**, 90128–5 (1988).

5. Yendiki, A., Koldewyn, K., Kakunoori, S., Kanwisher, N. & Fischl, B. Spurious group differences due to head motion in a diffusion MRI study. *NeuroImage* **88**, 79–90 (2014).
